# The role of cytoreductive radical prostatectomy and lymph node dissection in bone‐metastatic prostate cancer: A population‐based study

**DOI:** 10.1002/cam4.6292

**Published:** 2023-06-27

**Authors:** Tingshuai Zhai, Jinliang Ma, Yi Liu, Haitao Li, Yanli Peng, Wenmin Guo, Jiedong Jia, Xiaolin Wu, Huanrong Jiang, Jun Tian, Dongwen Wang

**Affiliations:** ^1^ Department of Urology, National Cancer Center/National Clinical Research Center for Cancer/Cancer Hospital & Shenzhen Hospital Chinese Academy of Medical Sciences and Peking Union Medical College Shenzhen 518116 China

**Keywords:** bone metastases, cytoreductive radical prostatectomy, lymph node dissection, prostate cancer, SEER‐Medicare, survival analysis

## Abstract

**Backgrounds:**

The role of cytoreductive radical prostatectomy (cRP) for bone‐metastatic prostate cancer (bmPCa) remains controversial. We aimed to figure out whether cRP and lymph node dissection (LND) can benefit bmPCa.

**Methods:**

11,271 PCa patients with bone metastatic burden from 2010 to 2019 were identified using SEER‐Medicare. Overall survival (OS) and cancer‐specific survival (CSS) rates were visualized using Kaplan–Meier plots. Multivariable Cox regression analyses were constructed to examine the effects of cRP and LND on survival, after stratifying to age, prostate specific antigen (PSA), clinical stages, Gleason score, metastatic burden, radiotherapy, and chemotherapy status.

**Results:**

317 PCa patients underwent cRP and cRP was increasingly performed for bmPCa from 2010 (2.2%) to 2019 (3.0%) (*p* < 0.05). In multi analyses, cRP was predisposed to a better OS or CSS in patients with age < 75, PSA < 98 ng/mL, bone‐only metastatic sites or patients not receiving chemotherapy (all *p* < 0.05). For the patients undergoing cRP, LND especially extended LND was associated with a better OS or CSS (all *p* < 0.05).

**Conclusions:**

cRP might benefit OS or CSS in young patients with low PSA and bone‐only metastatic sites not receiving chemotherapy. And a clear OS or CSS benefit of LND especially extended LND was observed in patients undergoing cRP.

## 1 BACKGROUND

Prostate cancer (PCa), accounting for 10% of the estimated cancer deaths in men, is increasingly threatening men's health.^1^ It is rare lethal in localized diseases (5‐year relative survival, > 99%). However, the survival drops precipitously when develops to distant stage (5‐year relative survival, 31%), which might result from the proclivity of aggressive PCa metastasizing to bone.^2^ In this case, a palliative multimodal therapeutic approach is regularly considered. Radical prostatectomy (RP), first described by Hugh Hampton Young in 1905, has evolved remarkably for treating PCa,^3^, ^4^ and now it is a proven cancer therapy for low‐moderate risk diseases. The operative concepts of RP have evolved rapidly since 21st century. Currently men with high‐risk diseases are often offered surgery for a favorable prognosis, which could not be imagined decades ago.^5^, ^6^

For bone‐metastatic prostate cancer (bmPCa), multidisciplinary therapy based on androgen deprivation therapy (ADT) is the standard treatment.^7^ As for the therapeutic role of cytoreductive radical prostatectomy (cRP), there were studies reporting its beneficial role for bmPCa especially for those with proven low‐volume or occult metastatic disease.^8^, ^9^ However, National Comprehensive Cancer Network (NCCN) and European Association of Urology (EAU) guidelines are silent on this topic.^10^ Characteristics including tolerance, age, comorbidities, PSA, grade, Gleason score, tumor stage, and metastatic burdens are often considered when determining whether to perform cRP. No previous study has investigate the efficiency of cRP on characteristic‐specific survival for bmPCa. Therefore, we carried out this research to verify the effect of cRP and lymph node dissection (LND) on overall survival (OS) and cancer‐specific survival (CSS) stratified to prostate specific antigen (PSA), age, clinical stages, Gleason score, metastatic burden, radiotherapy, and chemotherapy status.

## 2 METHODS

### 2.1 Study design

**FIGURE 1:**
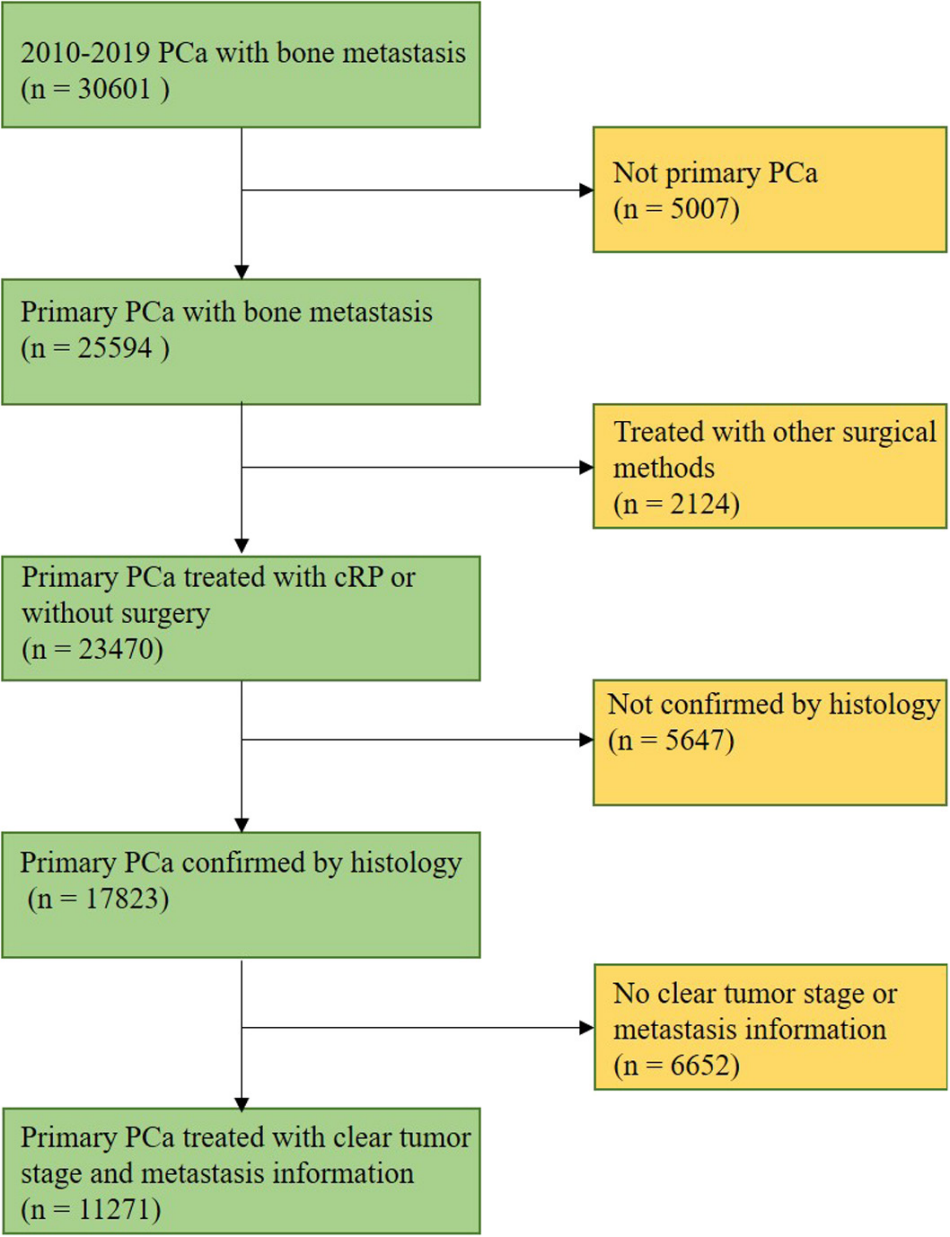
The flow chart describes the steps taken to identify 11,271 prostate cancer patients with bone metastases in the SEER database.

bmPCa patients were selected within the Surveillance, Epidemiology and End Results (SEER) Medicare database from 2010 to 2019. The eligibility criteria included: (1) bone metastases; (2) tumor sequence number labeled “one primary only”; (3) PCa confirmed by histology; (4) treated without surgery or with cRP (code 0 or 50); and (5) patients with clear clinical tumor stage and metastasis information. Finally, 11,271 patients with bmPCa were identified (Figure 1).

317 (2.8%) PCa patients receiving cRP were identified. When assessing the effect of LND, 311 of the 317 patients were selected since 6 patients with unclear LND status were excluded. LND with 1–12 or more than 12 regional lymph nodes (LNs) removed was defined as limited or extended.

### 2.2 Covariates

Covariate data were obtained from Medicare using codes. For surgical primary site, code 0 (no surgery) and 50 (radical prostatectomy) were identified. For scope of regional lymph node surgery, none, 1–12 regional LNs removed and more than 12 regional LNs removed were selected.

Patient‐level covariates included age, race (white, black, other), marital status (married, no/unknown), PSA, Gleason grade, clinical tumor stage (T1, T2, T3, T4), clinical lymph node stage (N0, N1), metastatic sites (bone only, multiple metastases), radiotherapy (yes, no), and chemotherapy (yes, no).

### 2.3 Outcomes

The primary outcomes were CSS and OS as coded by Medicare.

### 2.4 Statistical analysis

Continuous variables are reported as mean ± s.d. Student's *t*‐test, chi‐squared test or fisher's exact test was used as appropriate. Kaplan–Meier plots described OS and CSS curves. In multivariable analyses, covariates consisted of age, year of diagnosis, race (white vs. black vs. other), marital status (no/unknown vs. married), PSA (≤ 10 ng/mL vs. 10–50 ng/mL vs. 50–98 ng/mL vs. ≥ 98 ng/mL vs. Unknown), biopsy Gleason score (<7 vs. 3 + 4 vs. 4 + 3 vs. 8 vs. 9–10), T stage (T1 vs. T2 vs. T3 vs. T4), N stage (N0 vs. N1) (AJCC 7th ed), metastatic sites (Bone only vs. Multiple metastases), radiotherapy (yes vs. no) and chemotherapy (yes vs. no). The 95% CIs were calculated and *p* < 0.05 was considered statistically significant. SPSS (IBM SPSS Statistics 25) was used.

## 3 RESULTS

Among the 11,271 bmPCa patients within the SEER‐Medicare, 317 (2.8%) patients underwent cRP (Table 1). The majority were white race (73.0%), married (58.9%), bone only metastases (90.5%), no radiotherapy (74.2%), and no chemotherapy (81.6%). Furthermore, 1170 patients' PSA were 10 or less than 10 ng/mL (10.4%), 3105 with 10–50 ng/mL (27.5%), 1402 patients with 50–98 ng/mL (12.4%), 5078 patients with 98 or more than 98 ng/mL (45.1%), and 516 patients with unknown PSA (4.6%). 188 patients harbored Gleason score <7 (1.7%) versus 487 Gleason score 3 + 4 (4.3%) versus 894 Gleason score 4 + 3 (7.9%) versus 2491 Gleason score 8 (22.1%) versus 5742 Gleason score 9–10 (50.9%) versus 1469 unknown Gleason score (13.0). 3618 patients harbored T1 (32.1%) versus 4212 T2 (37.4%) versus 1845 T3 (16.4%) versus 1596 T4 (14.2%) stage and 7159 patients harbored N0 (63.5%) versus 4112 N1 (36.5%).

**TABLE 1:** Characteristics for prostate cancer patients with bone metastasis stratified by radical prostatectomy.

	Total^a^	No cRP^a^	cRP^a^	*p* value^b^
Characteristic	No. (%)	No. (%)	No. (%)	
Total	11,271 (100)	10,954 (97.2)	317 (2.8)	
Age (years)				< 0.001
< 55	818 (7.3)	772 (7.0)	46 (14.5)	
55–64	3092 (27.4)	2951 (26.9)	141 (44.5)	
65–74	3932 (34.9)	3821 (34.9)	111 (35.0)	
75–84	2581 (22.9)	2565 (23.4)	16 (5.0)	
≥ 85	848 (7.5)	845 (7.7)	3 (0.9)	
Year of diagnosis^c^	2015.1 ± 2.8	2015.0 ± 2.8	2015.5 ± 2.7	0.006
Race				0.005
White	8487 (75.3)	8227 (75.1)	260 (82.0)	
Black	1893 (16.8)	1861 (17.0)	32 (10.1)	
Other	891 (7.9)	866 (7.9)	25 (7.9)	
Marital status				< 0.001
Married	6640 (58.9)	6399 (58.4)	241 (76.0)	
No/unknown	4631 (41.1)	4555 (41.6)	76 (24.0)	
PSA				< 0.001
≤ 10 ng/mL	1170 (10.4)	1035 (9.4)	135 (42.6)	
10–50 ng/mL	3105 (27.5)	2977 (27.2)	128 (40.4)	
50–98 ng/mL	1402 (12.4)	1384 (12.6)	18 (5.7)	
≥ 98 ng/mL	5078 (45.1)	5061 (46.2)	17 (5.4)	
Unknown	516 (4.6)	497 (4.5)	19 (6.0)	
Gleason score				< 0.001
<7	188 (1.7)	176 (1.6)	12 (3.8)	
3 + 4	487 (4.3)	445 (4.1)	42 (13.2)	
4 + 3	894 (7.9)	846 (7.7)	48 (15.1)	
8	2491 (22.1)	2401 (21.9)	90 (28.4)	
9–10	5742 (50.9)	5633 (51.4)	109 (34.4)	
Unknown	1469 (13.0)	1453 (13.3)	16 (5.0)	
T stage				< 0.001
T1	3618 (32.1)	3612 (33.0)	6 (1.9)	
T2	4212 (37.4)	4139 (37.8)	73 (23.0)	
T3	1845 (16.4)	1624 (14.8)	221 (69.7)	
T4	1596 (14.2)	1579 (14.4)	17 (5.4)	
N stage				0.147
N0	7159 (63.5)	6967 (63.6)	192 (60.6)	
N1	4112 (36.5)	3987 (36.4)	125 (39.4)	
Metastatic sites				< 0.001
Bone only	10,203 (90.5)	9895 (90.3)	308 (97.2)	
Multiple metastases	1068 (9.5)	1059 (9.7)	9 (2.8)	
Radiotherapy				0.341
Yes	2903 (25.8)	2825 (25.8)	78 (24.6)	
No	8368 (74.2)	8129 (74.2)	239 (75.4)	
Chemotherapy				< 0.001
Yes	2076 (18.4)	2046 (18.7)	30 (9.5)	
No	9195 (81.6)	8908 (81.3)	287 (90.5)	

*Note*: T and N stage refer to AJCC cancer staging manual, 7th ed. cRP, cytoreductive radical prostatectomy.

^a^ With percentages in parentheses.

^b^ Fisher's exact test or chi‐squared test, except.

^c^ Student's *t* test.

Patients were divided into two group according to cRP recode: 10954 (97.2%) in non‐cRP group and 317 (2.8%) in cRP group. cRP was prone to be performed in young patients, recent years, white race, married, lower PSA, lower Gleason score, T3 stage, N1 stage, bone‐only metastases, and patients not undergoing chemotherapy (all *p* < 0.05) (Table 1). The cRP rate grew from 2.2% to 3.0% during the decade evidently. (Table S1; *p* = 0.046) (Figure S1). In multi analyses, low age, recent year of diagnosis, other race, married, and cRP were predisposed to a better OS or CSS while high PSA, high Gleason score, high T stage, N1, and multiple metastases were opposite (all *p* < 0.05). (Table S2).

**FIGURE 2:**
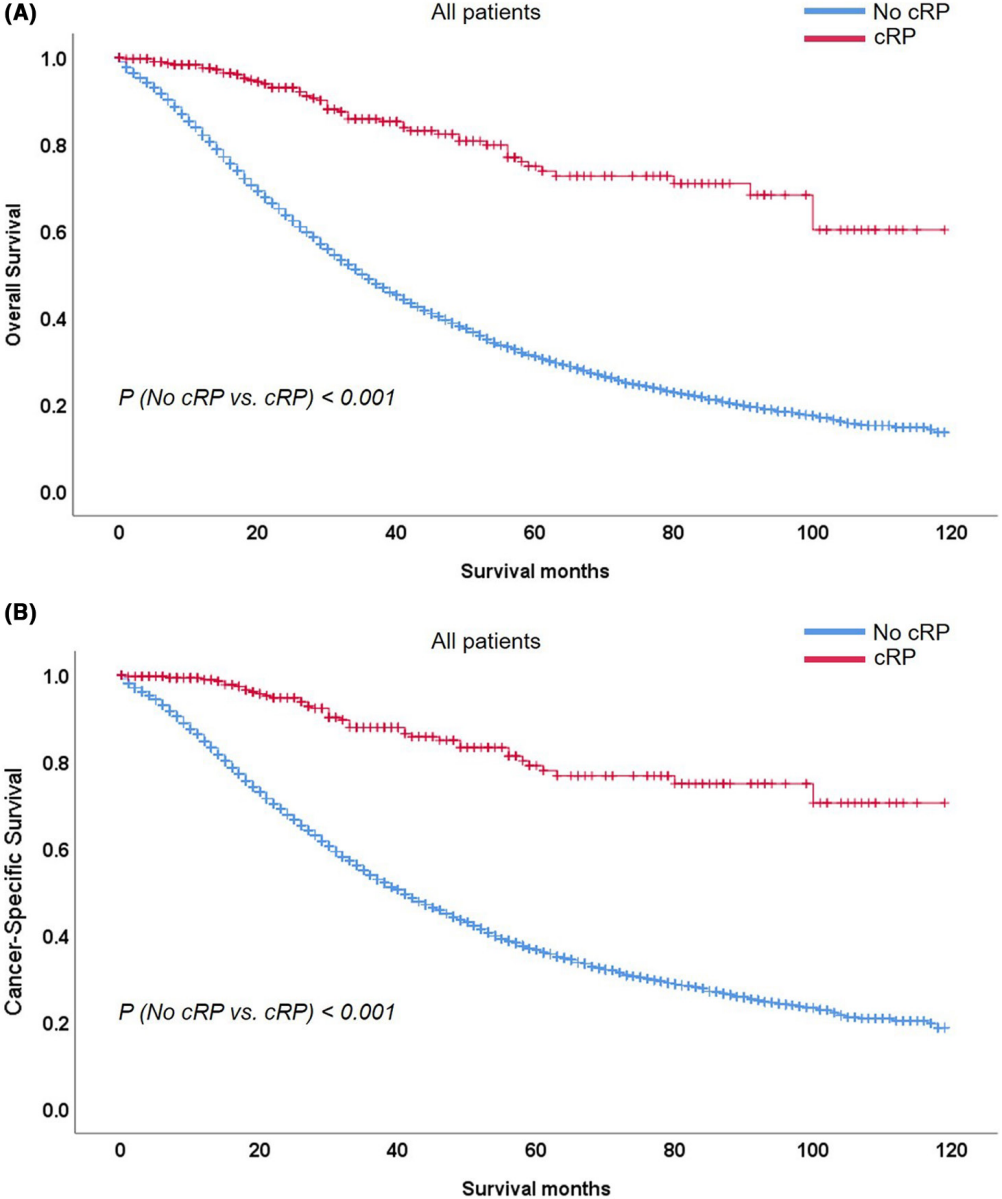
Kaplan–Meier plots depicting OS (A) and CSS (B) after stratification according to cRP status in 11,271 patients between 2010 and 2019 within the Surveillance, Epidemiology, and End Results database. cRP, cytoreductive radical prostatectomy; OS, overall survival; CSS, cancer‐specific survival.

**FIGURE 3:**
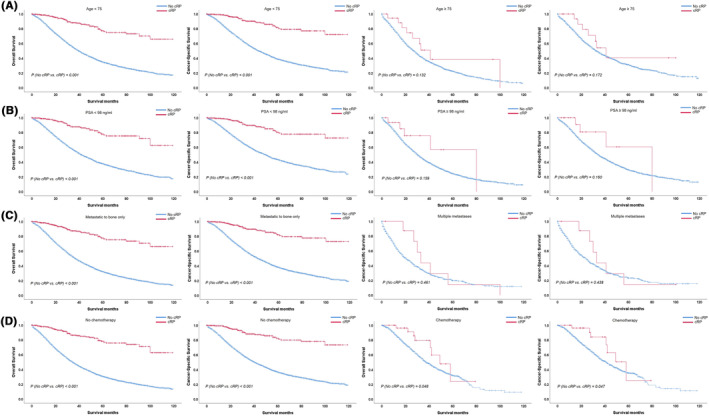
Kaplan–Meier plots depicting OS and CSS after stratification according to cRP status when stratified to age (A), PSA (B), metastatic sites (C) or chemotherapy status (D) in 11,271 patients. cRP, cytoreductive radical prostatectomy; CSS, cancer‐specific survival; OS, overall survival.

Table 2 showed the hazard ratios (HR) associated with cRP for survival. Patients undergoing cRP had lower HR for both OS (HR 0.39, 95%CI 0.30–0.52) and CSS (HR 0.35, 95%CI 0.25–0.48). Kaplan–Meier plots showed cRP was predisposed to a better OS or CSS (Figure 2). After stratified to age, PSA, T stage, N stage, Gleason score, metastatic sites, radiotherapy and chemotherapy status, the benefits of cRP on OS or CSS was obvious in patients aged ≤64 and 65–74 (OS: HR 0.33 and 0.43, respectively, *p* < 0.01; CSS: HR 0.30 and 0.34, respectively, *p* < 0.01) while disappeared in patients aged ≥75. Moreover, for PSA ≤10 ng/mL, 10–50 ng/mL and 50–98 ng/mL, the OS (HR 0.37, 0.38 and 0.12, respectively, *p* < 0.05) and CSS (HR 0.29, 0.40 and 0.14, respectively, *p* < 0.05) benefits were evident. For T2‐T3 or N0/N1 stages, cRP was associated with a better OS and CSS (all *p* < 0.001). The benefits of cRP on OS or CSS was seen in Gleason 3 + 4, 4 + 3, 9–10, and unknown group (OS: HR 0.34, 0.20, 0.42 and 0.42, respectively, *p* < 0.05; CSS: HR 0.10, 0.22, 0.39, and 0.33, respectively, *p* < 0.05). And cRP brought OS and CSS benefits in patients with bone‐only metastatic sites (*p* < 0.001) not patients with multiple metastases (*p* > 0.05). For patients receiving radiotherapy, cRP brought a OS or CSS benefit evidently (OS: HR 0.37, *p* < 0.01; CSS: HR 0.30, *p* < 0.01), for those treated without radiotherapy cRP brought an evident OS (HR 0.40, *p* < 0.01) and CSS (HR 0.36, *p* < 0.01) benefit. While, cRP brought an evident OS (HR 0.36, *p* < 0.001) and CSS (HR 0.31, *p* < 0.001) benefit for patients not undergoing chemotherapy, which disappeared in patients receiving chemotherapy. In Figure 3, Kaplan–Meier plots indicated that cRP brought an evident OS or CSS benefit in patients with age < 75, PSA < 98 ng/mL, bone‐only metastatic sites and no chemotherapy (all *p* < 0.001).

**TABLE 2:** Multivariable Cox regression analyses predicting overall survival and cancer‐specific survival stratified by cytoreductive radical prostatectomy.

	No cRP	cRP for OS	cRP for CSS
	HR (95% CI)	HR (95% CI)	HR (95% CI)
All patients	1.00 (Ref.)	0.39 (0.30–0.52)***	0.35 (0.25–0.48)***
Age			
≤ 64	1.00 (Ref.)	0.33 (0.22–0.49)***	0.30 (0.19–0.46)***
65–74	1.00 (Ref.)	0.43 (0.26–0.71)**	0.34 (0.19–0.63)**
≥ 75	1.00 (Ref.)	0.67 (0.34–1.29)	0.64 (0.30–1.36)
PSA			
≤ 10 ng/mL	1.00 (Ref.)	0.37 (0.23–0.60)***	0.29 (0.16–0.51)***
10–50 ng/mL	1.00 (Ref.)	0.38 (0.24–0.61)***	0.40 (0.25–0.65)***
50–98 ng/mL	1.00 (Ref.)	0.12 (0.02–0.88)*	0.14 (0.02–0.97)*
≥ 98 ng/mL	1.00 (Ref.)	0.74 (0.31–1.79)	0.65 (0.24–1.74)
Unknown	1.00 (Ref.)	0.70 (0.31–1.56)	0.57 (0.20–1.61)
T stage			
T1	1.00 (Ref.)	0.43 (0.11–1.73)	0.50 (0.13–2.01)
T2	1.00 (Ref.)	0.30 (0.17–0.55)***	0.22 (0.10–0.46)***
T3	1.00 (Ref.)	0.41 (0.28–0.59)***	0.38 (0.25–0.57)***
T4	1.00 (Ref.)	0.65 (0.27–1.58)	0.69 (0.28–1.69)
N stage			
N0	1.00 (Ref.)	0.38 (0.26–0.56)***	0.32 (0.20–0.49)***
N1	1.00 (Ref.)	0.39 (0.25–0.59)***	0.37 (0.23–0.58)***
Gleason score			
<7	1.00 (Ref.)	0.35 (0.04–2.76)	–
3 + 4	1.00 (Ref.)	0.34 (0.12–0.98)*	0.10 (0.01–0.75)*
4 + 3	1.00 (Ref.)	0.20 (0.05–0.81)*	0.22 (0.05–0.93)*
8	1.00 (Ref.)	0.64 (0.38–1.09)	0.63 (0.36–1.13)
9–10	1.00 (Ref.)	0.42 (0.28–0.65)***	0.39 (0.25–0.62)***
Unknown	1.00 (Ref.)	0.42 (0.19–0.90)*	0.33 (0.14–0.82)*
Metastatic sites			
Bone only	1.00 (Ref.)	0.38 (0.28–0.51)***	0.33 (0.23–0.46)***
Multiple metastases	1.00 (Ref.)	0.65 (0.30–1.42)	0.60 (0.26–1.39)
Radiotherapy			
Yes	1.00 (Ref.)	0.37 (0.20–0.69)**	0.30 (0.15–0.60)**
No	1.00 (Ref.)	0.40 (0.29–0.55)***	0.36 (0.25–0.51)***
Chemotherapy			
Yes	1.00 (Ref.)	0.91 (0.44–1.86)	0.81 (0.38–1.73)
No	1.00 (Ref.)	0.36 (0.26–0.49)***	0.31 (0.22–0.45)***

*Note*: Adjusted to age, year of diagnosis, race, marital status, PSA, Gleason score, tumor stage, metastatic sites, radiotherapy, and chemotherapy.

Abbreviations: 95% CI, 95% confidence interval; cRP, cytoreductive radical prostatectomy; CSS, cancer‐specific survival; HR, hazard ratio; OS, overall survival.

* *p* < 0.05

** *p* < 0.01

*** *p* < 0.001.

The 311 PCa patients undergoing cRP were divided into two groups according LND status (No LND vs. LND) and the results are showed in Table 3. LND was mainly performed in young, low PSA, high Gleason score, high grade, high T stage, N1 diseases, and bone‐only metastases (all *p* < 0.05). In multi analyses, LND including limited or extended LND was predisposed to a better OS (all *p* < 0.05) or CSS (all *p* < 0.05) in patients especially in T1/T2, N0, bone‐only metastases, and no radiotherapy. Multivariable cox analyses predicting OS for the 311 patients were described in Table S3 and the findings of LND on CSS were shown in Table S4. Details of the multiple metastases group are shown in Table S5. In Figure S2, LND (limited or extended LND) was predisposed to a better OS or CSS (all *p* < 0.05). And patient confirmed negative LN by LND had a better OS or CSS compared with those harboring >3 positive LNs or no LND (all *p* < 0.05).

**TABLE 3:** Characteristics for 311 prostate cancer patients with bone metastasis undergoing radical prostatectomy stratified by lymph node dissection.

	Total^a^	No LND^a^	LND^a^	*p* value^b^
Characteristic	No. (%)	No. (%)	No. (%)	
Total	311 (100)	47 (15.1)	264 (84.9)	
Age (years)^c^				
< 55	44 (14.1)	4 (8.5)	40 (15.2)	0.037
55–64	137 (44.1)	17 (36.2)	120 (45.5)	
65–74	111 (35.7)	20 (42.6)	91 (34.5)	
75–84	16 (5.1)	4 (8.5)	12 (4.5)	
≥ 85	3 (1.0)	2 (4.3)	1 (0.4)	
Year of diagnosis^c^	2015.5 ± 2.7	2015.0 ± 2.8	2015.6 ± 2.7	0.163
Race				0.744
White	255 (82.0)	38 (80.9)	217 (82.2)	
Black	31 (10.0)	4 (8.5)	27 (10.2)	
Other	25 (8.0)	5 (10.6)	20 (7.6)	
Marital status				0.499
Married	74 (23.8)	13 (27.7)	61 (23.1)	
No/Unknown	237 (76.2)	34 (72.3)	203 (76.9)	
PSA				0.001
≤ 10 ng/mL	132 (42.4)	21 (44.7)	111 (42.0)	
10–50 ng/mL	126 (40.5)	12 (25.5)	114 (43.2)	
50–98 ng/mL	18 (5.8)	1 (2.1)	17 (6.4)	
≥ 98 ng/mL	17 (5.5)	6 (12.8)	11 (4.2)	
Unknown	18 (5.8)	7 (14.9)	11 (4.2)	
Gleason score				< 0.001
<7	12 (3.9)	5 (10.6)	7 (2.7)	
3 + 4	40 (12.9)	10 (21.3)	30 (11.4)	
4 + 3	48 (15.4)	7 (14.9)	41 (15.5)	
8	89 (28.6)	9 (19.1)	80 (30.3)	
9–10	107 (34.4)	9 (19.1)	98 (37.1)	
Unknown	15 (4.8)	7 (14.9)	8 (3.0)	
T stage				< 0.001
T1	4 (1.3)	3 (6.4)	1 (0.4)	
T2	72 (23.2)	19 (40.4)	53 (20.1)	
T3	220 (70.7)	23 (48.9)	197 (74.6)	
T4	15 (4.8)	2 (4.3)	13 (4.9)	
N stage				< 0.001
N0	189 (60.8)	43 (91.5)	146 (55.3)	
N1	122 (39.2)	4 (8.5)	118 (44.7)	
Metastatic sites				0.013
Bone only	302 (97.1)	43 (91.5)	259 (98.1)	
Multiple metastases	9 (2.9)	4 (8.5)	5 (1.9)	
Radiotherapy				0.584
Yes	76 (24.4)	10 (21.3)	66 (25.0)	
No	235 (75.6)	37 (78.7)	198 (75.0)	
Chemotherapy				0.452
Yes	29 (9.3)	3 (6.4)	26 (9.8)	
No	282 (90.7)	44 (93.6)	238 (90.2)	

*Note*: T and N stage refer to AJCC Cancer Staging Manual, 7th ed. LND, lymph node dissection. Six patients of the 317 patients undergoing cRP were excluded due to the lack of LND information. LND, lymph node dissection.

^a^ With percentages in parentheses.

^b^ Fisher's exact test or chi‐squared test, except.

^c^ Student's *t* test.

## 4 DISCUSSION

In this study, cRP was only seen in 2.8% of the bmPCa from 2010 to 2019. Notably, urologists were increasingly willing to perform cRP for bmPCa patients (from 2.2% in 2010 to 3.0% in 2019), which indicated the increasing awareness of the therapeutic role of cRP for bmPCa patients. We observed that age, race, marital status, PSA, Gleason score, T/N stage, metastatic sites, and chemotherapy were significant factors contributing to the decision‐making of cRP. Using multivariable cox regression analyses, we identified low age, recent years, other races, married, and cRP were prone to a higher OS and similar findings were observed for CSS. However, elevated PSA, worse Gleason score, advanced T stage, N1, and multiple metastases were related to a worse OS or CSS. Moreover, in multivariable analyses, cRP was related to a preferable survival in patients with age ≤ 64 or 65–74, PSA < 10 or 10–50 or 50–98 ng/mL, T2/3 stage, N0/1, Gleason 3+4/4+3/9‐10/unknown, bone‐only metastatic sites or patients not receiving chemotherapy. For the patients undergoing cRP, LND especially extended LND was associated with a preferable survival. These findings suggest cRP might be acceptable in bmPCa especially for young patients with low PSA, bone‐only metastatic sites, and not receiving chemotherapy. Futhermore, LND especially extended LND should be considered when performing cRP.

There were researchers studying the role of cRP for bmPCa but a consensus seemed far off. Culp et al. reported that cRP or brachytherapy (*n* = 245) could bring a 5‐year OS (67.4% vs. 52.6%) and CSS (75.8% vs. 61.3%) benefit for M1a‐c PCa compared to no local treatment (*n* = 7811).^9^ Gratzke et al. reported a 5‐year OS of 55% for PCa undergoing cRP compared to 21% for those treated with no surgical resection.^11^ A study of 11 No patients with oligometastatic PCa performed by Gandaglia et al. showed a 7‐year CSS of 82% and cancer progression‐free survival of 45%.^12^ According to STAMPEDE trial^8^ and TROMBONE^13^ study, cRP might be an effective first step for PCa with a limited metastatic burden before a multidisciplinary approach. And our study showed patients receiving cRP had lower HR for both OS (HR 0.45, 95%CI 0.30–0.67) and CSS (HR 0.41, 95%CI 0.26–0.64), which are consistent with the aforementioned findings. However, an essential question in evaluating the feasibility of cRP for bmPCa is whether the detrimental effects can be offset by the benefits. Complications and functional outcomes are main concerns prior to surgery.^14^ While, several studies tested the safety of cRP for metastatic PCa and found no increased risk of complications or functional impairments.^15^, ^16^, ^17^ Thus, cRP might be a feasible and effective option for bmPCa. Whereas, it is still unclear which kind of patients may benefit from it. Rajwa et al. reviewed the recent prospective studies analyzing the survival of cRP in metastatic hormone‐sensitive prostate cancer and concluded that cRP is effective and safe in selected patients.^18^ However, they did not clarify the target population.

Therefore, we performed the multivariable analyses stratified to the characteristics including age, PSA, T stage, N stage, Gleason score, metastatic sites, radiotherapy and chemotherapy status, and we observed that the OS or CSS benefit was evident in patients with age < 75, PSA < 98 ng/mL, bone‐only metastases or patients not receiving chemotherapy. Based on these findings, we speculated that cRP might benefit OS or CSS for young patients with low PSA, bone‐only metastatic sites, and patients not receiving chemotherapy. The molecular biological mechanism supporting cRP's role remains being investigated. Increased expression of epithelial to mesenchymal transition (EMT) pathways were observed in the primary carcinoma at cRP despite 1‐year chemohormonal therapy.^19^ Src signal might be minimized by removing the primary tumor.^20^ Moreover, the source of disseminated tumor cells and the support of metastatic sites from the primary sites may be shut down by cRP.^21^, ^22^, ^23^

Currently evidences are insufficient to confirm the value of LND when performing cRP on bmPCa patients. First we performed a multi‐analysis for the 311 patients undergoing cRP and found LND especially extended LND may improve OS or CSS for them. Many attempts have been made to investigate the therapeutic role of LND but there is still no clear recommendation on this topic,^24^, ^25^, ^26^ largely due to the insufficient detectability of the multiplicity of nodal metastatic drainage and the LND templates needing to be improved.^27^, ^28^

Our study has limitations. The details such as the size and quantity of bone metastases were lacking. We defined 1–12 LNs removed as limited LND and 12 or more LNs removed as extended LND as a proxy as the extent of LND lacks standardization in SEER. Furthermore, only 317 of the 11,271 patients received cRP, which needs larger sample prospective randomized clinical trials (RCT) to validate. Up to now there have been nine ongoing RCTs (SWOG, IP2‐ATLANTA, SIMCAP, etc.) focusing on this topic,^29^ and we are looking forward to their findings.

To summarize, cRP might benefit OS or CSS in young patients with low PSA and bone‐only metastatic sites not receiving chemotherapy. And a clear OS or CSS benefit of LND especially extended LND was observed in patients undergoing cRP.

## AUTHOR CONTRIBUTIONS

**Ting‐Shuai Zhai:** Conceptualization (equal); data curation (equal); formal analysis (equal); investigation (equal); methodology (equal); project administration (equal). **Jinliang Ma:** Conceptualization (equal); data curation (equal); formal analysis (equal); investigation (equal); methodology (equal); project administration (equal). **Yi Liu:** Data curation (equal); formal analysis (equal); investigation (equal). **Haitao Li:** Data curation (equal); formal analysis (equal); investigation (equal); methodology (equal). **Yanli Peng:** Formal analysis (equal); investigation (equal); methodology (equal). **Wenmin Guo:** Data curation (equal); investigation (equal); methodology (equal). **Jiedong Jia:** Formal analysis (equal); investigation (equal); methodology (equal). **Xiaolin Wu:** Formal analysis (equal); investigation (equal). **Huanrong Jiang:** Conceptualization (equal); project administration (equal). **Jun Tian:** Conceptualization (equal); project administration (equal). **Dongwen Wang:** Conceptualization (equal); funding acquisition (equal); project administration (equal).

## CONFLICT OF INTEREST STATEMENT

The authors have no conflict of interest to declare.

## Supporting information


Data S1.

## Data Availability

Data sharing is not applicable to this article as no new data were created or analyzed in this study.
