# Supplementary material for: The role of cytoreductive radical prostatectomy and lymph node dissection in bone‐metastatic prostate cancer: A population‐based study
Source: Cancer Med. 2023 Jun 27;12(16):16697–706. doi: 10.1002/cam4.6292 (PMC10501265; doi:10.1002/cam4.6292)

**[The role of cytoreductive radical prostatectomy and lymph node dissection in bone-metastatic prostate cancer](https://www.zhangqiaokeyan.com/journal-foreign-detail/070405273582.html): A population-based study.**

Supplementary Table 1 Year of diagnosis for prostate cancer patients stratified by cytoreductive radical prostatectomy.

|  | Total* | No cRP* | cRP* | *P value*‡ |
| --- | --- | --- | --- | --- |
| Characteristic | No. (%) | No. (%) | No. (%) |  |
| Year of diagnosis |  |  |  | 0.046 |
| 2010 | 776 (100.0) | 759 (97.8) | 17 (2.2) |  |
| 2011 | 835 (100.0) | 812 (97.2) | 23 (2.8) |  |
| 2012 | 954 (100.0) | 936 (98.1) | 18 (1.9) |  |
| 2013 | 1006 (100.0) | 987 (98.1) | 19 (1.9) |  |
| 2014 | 1121 (100.0) | 1094 (97.6) | 27 (2.4) |  |
| 2015 | 1196 (100.0) | 1165 (97.4) | 31 (2.6) |  |
| 2016 | 1211 (100.0) | 1167 (96.4) | 44 (3.6) |  |
| 2017 | 1341 (100.0) | 1289 (96.1) | 52 (3.9) |  |
| 2018 | 1369 (100.0) | 1327 (96.9) | 42 (3.1) |  |
| 2019 | 1462 (100.0) | 1418 (97.0) | 44 (3.0) |  |

*With percentages in parentheses; ‡Spearman correlation analysis. cRP, cytoreductive radical prostatectomy.

Supplementary Table 2 Multivariable Cox regression analyses predicting overall survival and cancer-specific survival.

|  | OS | CSS |
| --- | --- | --- |
| Characteristic | HR (95% CI) | HR (95% CI) |
| Age (years) | 1.23 (1.20-1.26)*** | 1.16 (1.13-1.19)*** |
| Year of diagnosis | 0.95 (0.94-0.96)*** | 0.95 (0.94-0.96)*** |
| Race |  |  |
| White | 1.00 (Ref.) | 1.00 (Ref.) |
| Black | 1.04 (0.97-1.11) | 1.01 (0.94-1.09) |
| Other | 0.64 (0.57-0.72)*** | 0.62 (0.54-0.70)*** |
| Marital status |  |  |
| No/Unknown | 1.00 (Ref.) | 1.00 (Ref.) |
| Married | 0.85 (0.80-0.89)*** | 0.88 (0.84-0.94)*** |
| PSA |  |  |
| ≤ 10 ng/ml | 1.00 (Ref.) | 1.00 (Ref.) |
| 10-50 ng/ml | 1.05 (0.95-1.17) | 1.03 (0.92-1.15) |
| 50-98 ng/ml | 1.22 (1.09-1.38)** | 1.21 (1.07-1.37)** |
| ≥ 98 ng/ml | 1.43 (1.29-1.57)*** | 1.40 (1.26-1.55)*** |
| Unknown | 1.32 (1.14-1.53)*** | 1.27 (1.09-1.49)** |
| Gleason score |  |  |
| ＜7 | 1.00 (Ref.) | 1.00 (Ref.) |
| 3+4 | 1.47 (1.12-1.94)** | 1.79 (1.28-2.50)** |
| 4+3 | 1.49 (1.14-1.93)** | 1.91 (1.39-2.62)*** |
| 8 | 1.61 (1.26-2.06)*** | 2.03 (1.50-2.75)*** |
| 9-10 | 2.63 (2.06-3.36)*** | 3.46 (2.57-4.68)*** |
| Unknown | 2.92 (2.27-3.76)*** | 3.81 (2.81-5.19)*** |
| T stage |  |  |
| T1 | 1.00 (Ref.) | 1.00 (Ref.) |
| T2 | 1.02 (0.96-1.08) | 1.00 (0.93-1.07) |
| T3 | 0.86 (0.80-0.96)** | 0.89 (0.81-0.97)** |
| T4 | 1.30 (1.20-1.41)*** | 1.32 (1.21-1.45)*** |
| N stage |  |  |
| N0 | 1.00 (Ref.) | 1.00 (Ref.) |
| N1 | 1.06 (1.00-1.13)* | 1.09 (1.02-1.15)** |
| Metastatic sites |  |  |
| Bone only | 1.00 (Ref.) | 1.00 (Ref.) |
| Multiple metastases | 1.65 (1.51-1.79)*** | 1.71 (1.56-1.87)*** |
| Radiotherapy |  |  |
| No | 1.00 (Ref.) | 1.00 (Ref.) |
| Yes | 1.02 (0.96-1.08) | 1.04 (0.97-1.11) |
| Chemotherapy |  |  |
| No | 1.00 (Ref.) | 1.00 (Ref.) |
| Yes | 0.93 (0.87-1.01) | 0.97 (0.89-1.05) |
| cRP |  |  |
| No | 1.00 (Ref.) | 1.00 (Ref.) |
| Yes | 0.39 (0.30-0.52)*** | 0.35 (0.25-0.48)*** |

*P < 0.05, **P < 0.01, ***P < 0.001. Adjusted to age, year of diagnosis, race, marital status, PSA, Gleason score, tumor stage, metastatic sites, radiotherapy, chemotherapy and cytoreductive radical prostatectomy. HR, hazard ratio; 95% CI, 95% confidence interval. OS, overall survival; CSS, cancer-specific survival; cRP, cytoreductive radical prostatectomy.

Supplementary Table 3 Multivariable Cox regression analyses predicting overall survival for 311 patients undergoing cytoreductive radical prostatectomy stratified by lymph node dissection.

|  | No LND | LND | Limited LND | Extended LND |
| --- | --- | --- | --- | --- |
|  | HR (95% CI) | HR (95% CI) | HR (95% CI) | HR (95% CI) |
| All patients | 1.00 (Ref.) | 0.33 (0.14-0.76)** | 0.36 (0.16-0.82)* | 0.21 (0.07-0.62)** |
| Age |  |  |  |  |
| < 75 | 1.00 (Ref.) | 0.52 (0.19-1.42) | 0.55 (0.20-1.49) | 0.38 (0.11-1.33) |
| ≥ 75 | 1.00 (Ref.) | - | - | - |
| PSA |  |  |  |  |
| < 98 ng/ml | 1.00 (Ref.) | 0.32 (0.10-1.00) | 0.37 (0.12-1.16) | 0.20 (0.05-0.77)* |
| ≥ 98ng/ml | 1.00 (Ref.) | - | - | - |
| T stages |  |  |  |  |
| T1/T2 | 1.00 (Ref.) | 0.01 (0.00-0.51)** | 0.01 (0.00-0.30)** | - |
| T3/T4 | 1.00 (Ref.) | 0.34 (0.12-0.99)* | 0.39 (0.13-1.13) | 0.23 (0.06-0.84)* |
| N stage |  |  |  |  |
| N0 | 1.00 (Ref.) | 0.26 (0.10-0.72)** | 0.30 (0.11-0.85)* | 0.10 (0.00-0.16)** |
| N1 | 1.00 (Ref.) | 0.30 (0.02-3.93) | 0.30 (0.02-3.96) | 0.29 (0.02-4.58) |
| Gleason score |  |  |  |  |
| ≤ 7 | 1.00 (Ref.) | 0.65 (0.05-8.33) | 0.95 (0.08-10.91) | - |
| > 7 | 1.00 (Ref.) | 0.75 (0.18-3.14) | 0.77 (0.19-3.21) | 0.64 (0.12-3.21) |
| Metastatic sites |  |  |  |  |
| Bone only | 1.00 (Ref.) | 0.34 (0.14-0.79)* | 0.37 (0.16-0.86)* | 0.21 (0.07-0.67)** |
| Multiple metastases | 1.00 (Ref.) | - | - | - |
| Radiotherapy |  |  |  |  |
| Yes | 1.00 (Ref.) | 0.24 (0.04-1.45) | 0.23 (0.03-1.60) | 0.26 (0.02-3.38) |
| No | 1.00 (Ref.) | 0.33 (0.11-1.05) | 0.34 (0.11-1.07) | 0.21 (0.05-0.91)* |
| Chemotherapy |  |  |  |  |
| Yes | 1.00 (Ref.) | - | - | - |
| No | 1.00 (Ref.) | 0.48 (0.19-1.2) | 0.53 (0.21-1.34) | 0.31 (0.09-1.05) |

*P < 0.05, **P < 0.01, ***P < 0.001. Adjusted to age, year of diagnosis, race, marital status, PSA, Gleason score, tumor stage, metastatic sites, radiotherapy and chemotherapy. HR, hazard ratio; 95% CI, 95% confidence interval. LND, lymph node dissection.

Supplementary Table 4 Multivariable Cox regression analyses predicting cancer-specific survival for 311 patients undergoing radical prostatectomy stratified by lymph node dissection.

|  | No LND | LND | Limited LND | Extended LND |
| --- | --- | --- | --- | --- |
|  | HR (95% CI) | HR (95% CI) | HR (95% CI) | HR (95% CI) |
| All patients | 1.00 (Ref.) | 0.21 (0.08-0.57)** | 0.23 (0.09-0.62)** | 0.15 (0.04-0.54)** |
| Age |  |  |  |  |
| < 75 | 1.00 (Ref.) | 0.41 (0.12-1.47) | 0.43 (0.12-1.53) | 0.36 (0.08-1.61) |
| ≥ 75 | 1.00 (Ref.) | - | - | - |
| PSA |  |  |  |  |
| < 98 ng/ml | 1.00 (Ref.) | 0.29 (0.08-1.04) | 0.32 (0.09-1.17) | 0.21 (0.05-0.97)* |
| ≥ 98ng/ml | 1.00 (Ref.) | - | - | - |
| T stages |  |  |  |  |
| T1/T2 | 1.00 (Ref.) | 0.01 (0.00-0.43)* | 0.01 (0.00-0.45)** | - |
| T3/T4 | 1.00 (Ref.) | 0.21 (0.06-0.72)* | 0.22 (0.06-0.77)* | 0.18 (0.04-0.81)* |
| N stage |  |  |  |  |
| N0 | 1.00 (Ref.) | 0.15 (0.04-0.54)** | 0.12 (0.03-0.53)** | 0.00 (0.00-0.03)** |
| N1 | 1.00 (Ref.) | 0.36 (0.03-4.83) | 0.35 (0.03-4.87) | 0.40 (0.03-6.37) |
| Gleason score |  |  |  |  |
| ≤ 7 | 1.00 (Ref.) | - | - | - |
| > 7 | 1.00 (Ref.) | 0.69 (0.15-3.15) | 0.69 (0.15-3.18) | 0.69 (0.12-3.94) |
| Metastatic sites |  |  |  |  |
| Bone only | 1.00 (Ref.) | 0.25 (0.09-0.71)* | 0.27 (0.09-0.77)* | 0.18 (0.05-0.68)* |
| Multiple metastases | 1.00 (Ref.) | - | - | - |
| Radiotherapy |  |  |  |  |
| Yes | 1.00 (Ref.) | 0.25 (0.03-2.16) | 0.26 (0.02-2.96) | 0.24 (0.02-3.51) |
| No | 1.00 (Ref.) | 0.25 (0.06-0.99)* | 0.25 (0.06-1.01) | 0.19 (0.03-1.09) |
| Chemotherapy |  |  |  |  |
| Yes | 1.00 (Ref.) | - | - | - |
| No | 1.00 (Ref.) | 0.34 (0.11-1.04) | 0.36 (0.11-1.13) | 0.28 (0.07-1.11) |

*P < 0.05, **P < 0.01, ***P < 0.001. Adjusted to age, year of diagnosis, race, marital status, PSA, Gleason score, tumor stage, metastatic sites, radiotherapy and chemotherapy. HR, hazard ratio; 95% CI, 95% confidence interval. LND, lymph node dissection.

Supplementary Table 5 Details of the multiple metastases group.

|  | No. (%) |
| --- | --- |
| Total | 1068 (100.0) |
| Bone + liver | 243 (22.8) |
| Bone + lung | 642 (60.1) |
| Bone + brain | 47 (4.4) |
| Bone + liver + lung | 116 (10.9) |
| Bone + liver +brain | 1 (0.1) |
| Bone + lung + brain | 7 (0.6) |
| Bone + liver + lung + brain | 12 (1.1) |

Supplementary Figure 1 Trend of cRP rate from 2010 to 2019.

cRP, cytoreductive radical prostatectomy.

Supplementary Figure 2 Kaplan-Meier plots depicting OS and CSS stratified according to LND status including no LND, limited LND and extended LND (A) or the number of positive lymph nodes(B) in 311 patients undergoing cRP. LND, lymph node dissection; OS, overall survival; CSS, cancer-specific survival; cRP, cytoreductive radical prostatectomy.


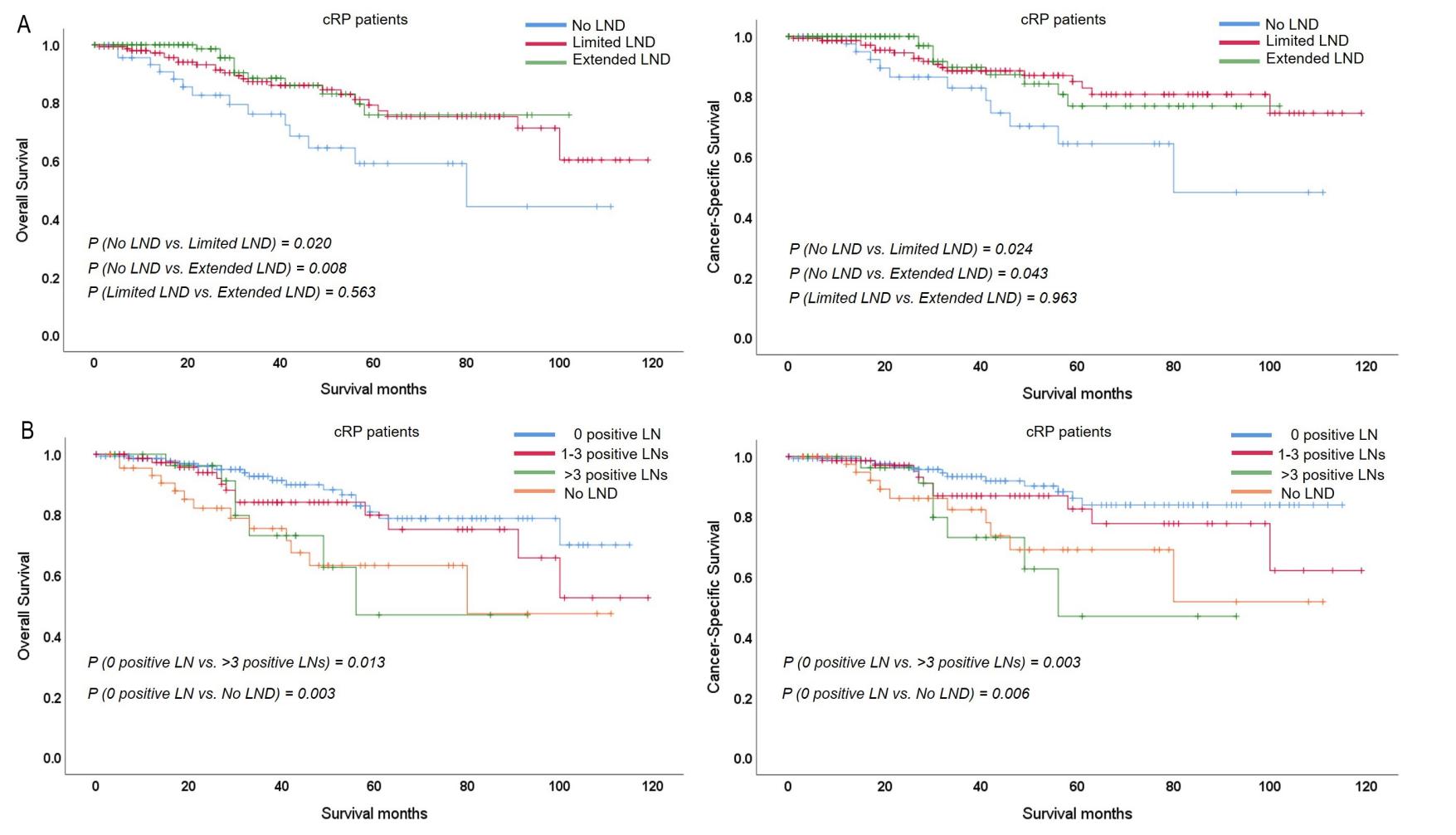

Supplement: Supplementary file 1 — Data S1. [file CAM4-12-16697-s001.docx]
